# Targeting Integrins for Cancer Therapy - Disappointments and Opportunities

**DOI:** 10.3389/fcell.2022.863850

**Published:** 2022-03-09

**Authors:** Cecilia Bergonzini, Kim Kroese, Annelien J. M. Zweemer, Erik H. J. Danen

**Affiliations:** Leiden Academic Center for Drug Research, Leiden University, Leiden, Netherlands

**Keywords:** integrin, extracellular matrix, therapy, clinical trial, cancer

## Abstract

Integrins mediate adhesive interactions between cells and their environment, including neighboring cells and extracellular matrix (ECM). These heterodimeric transmembrane receptors bind extracellular ligands with their globular head domains and connect to the cytoskeleton through multi-protein interactions at their cytoplasmic tails. Integrin containing cell–matrix adhesions are dynamic force-responsive protein complexes that allow bidirectional mechanical coupling of cells with their environment. This allows cells to sense and modulate tissue mechanics and regulates intracellular signaling impacting on cell faith, survival, proliferation, and differentiation programs. Dysregulation of these functions has been extensively reported in cancer and associated with tumor growth, invasion, angiogenesis, metastasis, and therapy resistance. This central role in multiple hallmarks of cancer and their localization on the cell surface makes integrins attractive targets for cancer therapy. However, despite a wealth of highly encouraging preclinical data, targeting integrin adhesion complexes in clinical trials has thus far failed to meet expectations. Contributing factors to therapeutic failure are 1) variable integrin expression, 2) redundancy in integrin function, 3) distinct roles of integrins at various disease stages, and 4) sequestering of therapeutics by integrin-containing tumor-derived extracellular vesicles. Despite disappointing clinical results, new promising approaches are being investigated that highlight the potential of integrins as targets or prognostic biomarkers. Improvement of therapeutic delivery at the tumor site via integrin binding ligands is emerging as another successful approach that may enhance both efficacy and safety of conventional therapeutics. In this review we provide an overview of recent encouraging preclinical findings, we discuss the apparent disagreement between preclinical and clinical results, and we consider new opportunities to exploit the potential of integrin adhesion complexes as targets for cancer therapy.

## Introduction

### Integrin Structure

Integrins represent a family of transmembrane adhesion receptors, facilitating the adhesive connection between cells and their surrounding extracellular matrix (ECM) or neighboring cells (Takada et al., 2007; Barczyk et al., 2009; Kadry and Calderwood, 2020). They comprise a group of heterodimeric proteins generated by non-covalent association of an *a*- and a *ß*-subunit (Ginsberg, 2014). Both subunits are classified as type 1 transmembrane proteins, composed of a rather large extracellular domain and a relatively small transmembrane- and intracellular region (Calderwood, 2004; Ginsberg, 2014). The globular head domain creates a binding site for extracellular ligands while the short cytoplasmic tails interact with a cluster of associated proteins that ultimately connects to the cytoskeleton. In total there are 18 α-and eight *ß*-subunits, generating 24 different heterodimers, known to be expressed in humans (Calderwood, 2004). This variety in combinations allows integrins to interact with—and respond to a broad range of ligands, including insoluble ECM proteins, matricellular proteins, cell surface proteins, and soluble proteins (Alday-Parejo et al., 2019). Several recognition motifs for integrin-binding have been identified. The Arg-Gly-Asp (RGD) motif is recognized by eight different integrins and has been found in a plethora of molecules ranging from ECM proteins to growth factors to coats of microorganisms.

### Integrin Function

Integrin transmembrane receptors execute two core functions: they mediate adhesion of cells to the ECM or neighboring cells, and they engage in transduction of signals received from the microenvironment. Integrin-mediated cell adhesion is dynamic: flexibility in integrin conformation allows a balance between active (open; high affinity) and inactive (closed; low affinity) states. The active state is regulated by interaction of the intracellular adaptor proteins talin and kindlin with the *ß*-subunit cytoplasmic tail and is further stabilized by interaction with ligand at the extracellular integrin head domain (Calderwood, 2004; Sun et al., 2019). Moreover, firm cell adhesion requires integrins to cluster in cell adhesion complexes that connect to the cytoskeleton.

Integrin-mediated cell adhesion controls many aspects of cell behavior including survival, proliferation, metabolism, differentiation, as well as cell shape and motility (Huveneers and Danen, 2009). Several mechanisms of such outside-in signaling have been proposed. First, integrins allow cells to interact with the ECM in which soluble growth factors such as VEGF, TGFβ and many others are concentrated, modified, and presented to cells (Hynes, 2009). Second, integrins can directly bind and activate growth factors such that they can stimulate their cognate receptors, a process currently established for activation of TGFβ by αvβ6 and αvβ8 (Margadant and Sonnenberg, 2010). Third, integrin engagement and clustering can lead to local activation of receptors for soluble ligands such as EGF, PDGF, and others, often involving receptor crosstalk via Src family kinases (Ivaska and Heino, 2011; Brizzi et al., 2012). Fourth, the dynamic intracellular complex of adaptor and signaling proteins that couples integrins to the cytoskeleton allows 1) local signaling through GTPases and kinases and 2) sensing of- and responding to mechanical aspects of the microenvironment by mechanoresponsive interactions (Huveneers and Danen, 2009; Kechagia et al., 2019).

### Integrins in Cancer

Dysregulation of integrin expression on cancer cells has been extensively studied in cell culture and animal models and shown to provide therapeutic opportunities for arresting tumor growth, reducing resistance to chemo-or radiotherapy, or attenuating invasion and metastasis. Studies using genetically engineered mouse models or using human tumor cells transplanted in immune deficient mice have extensively shown that deletion of integrins in cancer cells or preventing integrin function with blocking antibodies or peptides could interfere with tumor growth, metastasis, and resistance to chemo- or radiotherapy (Juliano and Varner, 1993; Danen, 2005; Desgrosellier and Cheresh, 2010; Hamidi and Ivaska, 2018; Cooper and Giancotti, 2019). For the large family of β1 integrins, dual roles have been identified in growth versus metastasis, indicating that caution is warranted for their application as therapeutic targets (Ramirez et al., 2011; Moran-Jones et al., 2012; Truong et al., 2014; Moritz et al., 2021). Integrins such as αvβ3, αvβ5, and α5β1, are not only expressed on tumor cells but are also induced on endothelial cells during the process of angiogenesis (Friedlander et al., 1995; Avraamides et al., 2008). These integrins have indeed been shown to serve as targets for anti-angiogenic therapies in cancer, although the mode of action of anti-angiogenic drugs targeting integrins remains enigmatic (Friedlander et al., 1995; Hynes, 2002; Alavi and Cheresh, 2008; Avraamides et al., 2008).

Recent studies have added a range of novel emerging cancer-related processes that require the participation of integrins, including the establishment of a pre-metastatic niche, epithelial-to-mesenchymal transition (EMT), metabolic rewiring, cancer cell stemness and dormancy (Barkan et al., 2010; Goel et al., 2014; Seguin et al., 2015; Ata and Antonescu, 2017; Ji et al., 2020; Park and Nam, 2020; Winkler et al., 2020; Coban et al., 2021). The involvement of integrin αvβ6 in activation of TGFβ was recently connected to SOX4 mediated cancer immune evasion: αvβ6 blocking antibodies could inhibit SOX4 expression and sensitize mouse models for triple negative breast cancer to T cell mediated killing in response to immune checkpoint inhibitors (Bagati et al., 2021). Integrin αvβ8, which can also activate TGFβ, represents a target expressed on immune cells for modulating anti-tumor immunity. I.e., αvβ8 blocking antibodies or specific depletion of integrin αvβ8 from the surface of CD4^+^CD25^+^ regulatory T cells could attenuate TGFβ mediated inhibition of CD8^+^ T cells and thereby restore tumor killing capacity of CD8^+^ T cells and synergizing with radio- or immune therapy (Dodagatta-Marri et al., 2021).

The expression of integrins on the cell-surface and their apparent role in several cancer related processes makes them appealing targets for the development of cancer therapies. However, despite the abundance of promising preclinical data, integrin targeting therapies in clinical studies have thus far largely failed to deliver. Notably, although not within the scope of this review, components of the integrin signaling complexes represent additional targets in cancer. For example, focal adhesion kinase (FAK) is overexpressed or activated in multiple cancers and supports tumor cell proliferation, migration, and therapy resistance. Small molecule inhibitors targeting FAK, such as defactinib, GSK2256098, VS-6063, and BI 853520, are currently being investigated in several clinical trials, mostly in combination with other agents (Mohanty et al., 2020; Dawson et al., 2021). Src is another interesting target associated with integrin signaling. Dasatinib, a Src inhibitor, showed efficacy when combined with docetaxel in castration-resistant prostate cancer patients (Araujo et al., 2012) (NCT00439270), and was more effective than imatinib in Pediatric Philadelphia Chromosome–Positive Acute Lymphoblastic Leukemia (Shen et al., 2020). On the other hand, dasatinib monotherapy failed to meet expectations in patients with recurrent glioblastoma (Lassman et al., 2015) or in patients with locally advanced or stage IV mucosal, acral, or vulvovaginal melanoma (Kalinsky et al., 2017). The challenges of targeting Src family proteins were recently reviewed by Martellucci and others (Martellucci et al., 2020). Integrins interact with many other cytoplasmic proteins, which are being investigated for their potential as therapeutic targets, however these have not yet been translated to the clinic (Cabodi et al., 2010; Bachmann et al., 2019).

In this review we focus on integrins as drug targets in cancer and discuss the apparent disagreement between preclinical and clinical results, we provide an overview of new encouraging preclinical findings and consider new opportunities to exploit the potential of integrin adhesion complexes in the effective treatment of cancer.

## Finalized Clinical Trials Exploring Integrin Therapeutics

Monoclonal antibodies and synthetic RGD peptides have been used in clinical trials to target integrins (Li M. et al., 2021). These drugs typically block integrin function by occupying the ligand binding site. Integrin blocking antibodies previously showed efficacy in different diseases, such as multiple sclerosis, thrombosis prevention after percutaneous coronary intervention, ulcerative colitis and Chron’s disease (Ley et al., 2016). Moreover, in multiple preclinical studies, inhibition of αvβ3, αvβ5 or β1 integrins prevented tumor angiogenesis, reduced tumor growth and limited metastatic spread, supporting the translation of these antibodies and blocking peptides into the clinic for cancer therapy (Mitjans et al., 2000; Trikha et al., 2004; Khalili et al., 2006; Danen, 2013). Despite promising preclinical results, such therapeutics did not make it to the market. Therapeutic safety was often not the bottleneck for integrin targeting therapeutics. The major drawback was their lack of efficacy (Table 1).

**TABLE 1:** Overview of failed or terminated major clinical trials for the assessment of integrin targeting therapeutics in cancer.

Clinical trial identifier	Phase	Name therapeutic	Type therapeutic	Target integrin	Combination therapy with	Condition	Result	Mode of action
NCT01360840	II	Abituzumab (EMD525797)	Antibody	αV	—	Metastatic Castration-Resistant Prostate cancer	PFS not significantly different	Blocks cell adhesion
NCT01008475	I/II	Abituzumab (EMD525797)	Antibody	αV	Cetuximab Irinotecan	Metastatic colorectal cancer	PFS not significantly different	Blocks cell adhesion
NCT00246012	II	Intetumumab (CNTO 95)	Antibody	αV	Dacarbazine	Stage IV Melanoma	PFS not significantly different	Blocks ligand binding site
NCT00537381	II	Intetumumab (CNTO 95)	Antibody	αV	Docetaxel Prednisone	Metastatic Hormone Refractory Prostate Cance	All efficacy endpoints better in placebo	Blocks ligand binding site
	II	Vitaxin (MEDI-523)	Antibody	αVβ3	—	Metastatic cancers	No tumor regression	Blocks ligand binding site
	II	Etaracizumab (MEDI-522, Abegrin)	Antibody	αVβ3	Dacarbazine	Stage IV metastatic melanoma	PFS not significantly different	Blocks ligand binding site
NCT00842712, NCT00121238, NCT00705016	II	Cilengitide (EMD 121974)	Inhibitory peptide	αVβ3/αVβ5	Multiple combinations	Multiple cancers	No benefits compared to standard of care	Blocks ligand binding site
NCT00689221	III	Cilengitide (EMD 121974)	Inhibitory peptide	αVβ3/αVβ5	Temozolomide + Radiotherapy	Newly Diagnosed Glioblastoma	Median OS not significantly different	Blocks ligand binding site
NCT00401570, NCT00654758, NCT00516841, NCT00635193, NCT00369395, NCT00100685	I/II	Volociximab (M200)	Antibody	αVβ1	Alone or in combinations with standard of care	Metastatic Pancreatic Cancer, Non-Small Cell Lung Cancer, Ovarian and Peritoneal cancer, Renal cell carcinoma	Partial or no significant effects	Blocks ligand binding site
NCT00675428	II	Natalizumab	Antibody	VLA-4, (α4)	—	Multiple myeloma	Terminated due to low enrollment	Allosteric inhibition
NCT00131651, NCT00352313	I/II	ATN-161	Small peptide antagonist	α5β1	Alone or in combinations	Glioma, renal cancer and other solid tumors	No therapeutic benefits	Blocks ligand binding site; prevents interaction with fibronectin synergy site
NCT01313598	I	GLPG0187	Non-peptide Integrin antagonist	Arg-Gly-Asp (RGD)-binding integrins	—	Solid tumors	No effect	Blocks ligand binding site

The majority of integrin directed therapeutics in clinical trials involve antibodies or peptides targeting αv-integrins and these have thus far failed to show benefit for cancer patients. The integrin αv antibody abituzumab was used in a phase II trial to treat patients with metastatic castration-resistant prostate cancer (Hussain et al., 2016) (NCT01360840). Even though a reduction in prostate cancer associated-bone lesion development was observed in the antibody treated group of patients, the primary endpoint of progression free survival (PFS) was not significantly extended. Interestingly, the addition of abituzumab to the standard of care did show some beneficial effect on the overall survival of a subset of metastatic colorectal carcinoma patients (Élez et al., 2015; Laeufle et al., 2018) (NCT01008475). Another phase II αv-targeting study illustrated that a combination treatment of dacarbazine with the monoclonal αv-antibody intetumumab did not enhance treatment efficacy over monotreatment in patients with stage IV melanoma (O'Day et al., 2011) (NCT00246012). Testing this antibody in a phase II trial with prostate cancer patients did not improve outcome either (Heidenreich et al., 2013) (NCT00537381). Antibodies specific for αvβ3 integrin have been extensively evaluated in clinical trials as well (Li M. et al., 2021). In a phase I trial, the αvβ3-antibody vitaxin failed to show benefit for patients with metastatic solid tumors (Posey et al., 2001). The additional effect of the αvβ3-antibody etaracizumab was assessed on top of dacarbazine treatment in stage IV melanoma patients (Hersey et al., 2010), however no significant differences in the time to progression (TTP) or PFS were observed. Several phase II trials have explored efficacy of the αvβ3/αvβ5-selective function blocking peptide cilengitide for treatment of solid tumors alone or in combination with other therapies, but results were not encouraging (Alva et al., 2012; Vermorken et al., 2014; Vansteenkiste et al., 2015; Alday-Parejo et al., 2019) (NCT00842712, NCT00121238, NCT00705016). Likewise, cilengitide failed to improve therapeutic efficacy in combination with standard of care for patients with newly diagnosed glioblastoma in a phase III trial (Stupp et al., 2014) (NCT00689221).

Other integrins that have been targeted include α5β1. Unfortunately, phase I and II trials using the small peptide antagonist of integrin α5β1 ATN-161 have thus far not shown benefit for glioma patients or in other solid tumors (Cianfrocca et al., 2006) (NCT00131651, NCT00352313). Similarly, the combination treatment of gemcitabine with the α5β1 chimeric monoclonal antibody volociximab did not show any additional treatment efficacy over gemcitabine monotreatment in metastatic pancreatic cancer patients in a phase II trial (Evans et al., 2007) (NCT00401570). Moreover, volociximab efficacy was not encouraging in peritoneal, ovarian, non-small cell lung cancer or melanoma (Figlin et al., 2006; Barton, 2008; Vergote et al., 2009; Bell-McGuinn et al., 2011; Besse et al., 2013) (NCT00401570, NCT00654758, NCT00516841, NCT00635193, NCT00369395, NCT00100685). Natalizumab, an antibody targeting α4β1 (VLA-4) has shown promising clinical results in autoimmune related diseases such as multiple sclerosis and Crohn’s disease (Rudick et al., 2013; McLean and Cross, 2016). However, a phase 1/2, two-arm dose-finding study of natalizumab for relapsed or refractory Multiple Myeloma, was unfortunately terminated due to insufficient patient enrolment (NCT00675428). Among the therapeutics discussed so far, natalizumab is the only one not targeting the ligand binding site. Instead, it acts through allosteric interactions (Yu et al., 2013). Further exploring such alternative forms of integrin receptor pharmacology may lead to new and more effective treatments (Slack et al., 2022).

## Ongoing Clinical Trials Exploring Integrin Therapeutics

As discussed, clinical trials of αv-integrin inhibitors or drugs targeting other integrins have thus far not been encouraging. Other approaches are now being explored in new clinical trials (Table 2).

**TABLE 2:** Overview of planned or ongoing clinical trials for the assessment of integrin targeting therapeutics in cancer.

Clinical trial identifier	Phase	Name therapeutic	Type therapeutic	Target integrin	Combination therapy with	Condition	Result
NCT05085548	I	ProAgio	Cytotoxic Protein	*α*Vβ3	—	Pancreatic cancer/Solid tumors	Recruiting
NCT04389632	I	SGN-B6A	Antibody-Drug Conjugate	*β*6	—	Solid tumors	Recruiting
NCT04608812	I	OS2966	First-in-class monoclonal Ab	*β*1	—	High-grade Glioma	Recruiting
NCT04508179	I	7HP349	Allosteric Integrin activation	*α*L*β*2/*α*4*β*1	—	Healthy subjects	Recruiting
NCT03517176	I	CEND-1	First-in-class iRGD	*α*V	Gemcitabine/Nab-Paclitaxel	Pancreatic cancer	PFS

A phase I trial aims to treat patients with previously treated pancreatic cancer or other solid tumors with the anti-αvβ3 protein, ProAgio (NCT05085548). ProAgio binds αvβ3 outside the classical ligand-binding site. Instead of blocking ligand binding, it triggers recruitment and activation of caspase 8, resembling a mechanism previously associated with unligated integrins (Stupack et al., 2001; Turaga et al., 2016). This may lead to apoptosis in tumor cells, endothelial cells, and cancer-associated fibroblasts with increased expression of αvβ3. Subsequently, this can result in a reduction of the stroma density of pancreatic cancer patients increasing access of conventional anti-cancer therapeutics to the tumor.

In a planned phase I trial, the safety, tolerability and efficacy of the integrin β6 targeting antibody-drug conjugate SGN-B6A will be studied in patients with advanced solid tumors. SGN-B6A consists of an antibody targeting integrin β6 conjugated with monomethyl auristatin E, an antimitotic agent that induces apoptosis by binding to tubulin (Patnaik et al., 2021) (NCT04389632). A randomized phase II trial, planned at the end of 2021 will study efficacy of a tumor penetrating iRGD peptide, CEND-1, in combination with gemcitabine and nab-paclitaxel in patients with metastatic pancreatic cancer. The first-in-class agent CEND-1 binds tumor cells and enhances delivery of co-administered anti-cancer agents. In a recently completed phase I clinical trial the safety and efficacy of CEND-1 was already explored (Dean et al., 2020; Dean et al., 2021) (NCT03517176). Based on the trial data, the combination treatment was regarded as safe. Importantly, efficacy of this treatment exceeded the efficacy of the mono-treatments, with ongoing progression free survival of some patients.

A first-in-class humanized and de-immunized monoclonal antibody, OS2966, that targets the β1 integrin subunit is tested in patients with high-grade glioma (Nwagwu et al., 2021) (NCT04608812). Considering that OS2966 targets the entire family of β1 containing integrins, toxicity may be an issue. Interestingly, this trial will make use of real time imaging. By adding gadolinium contrast to the OS2966 antibody, therapeutic distribution can be visualized using MRI. The additional collection of tissue specimens planned before and after treatment will provide better knowledge on the presence of any predictive biomarkers. In October 2021 a phase I trial finished, in which the safety, tolerability and pharmacokinetics (PK) of the allosteric integrin activator 7HP349 was studied in healthy male subjects (NCT04508179). Interestingly, in contrast to most integrin targeting therapeutics, this small molecule is designed to enhance integrin activity. Binding of 7HP349 should cause the activation of the αLβ2 and α4β1 integrins on immune cells, thereby enhancing an immune response. Results of this study remain to be published.

## Why Have Integrin-Targeted Therapeutics FAILED TO Achieve Clinical Efficacy Thus Far?

**FIGURE 1:**
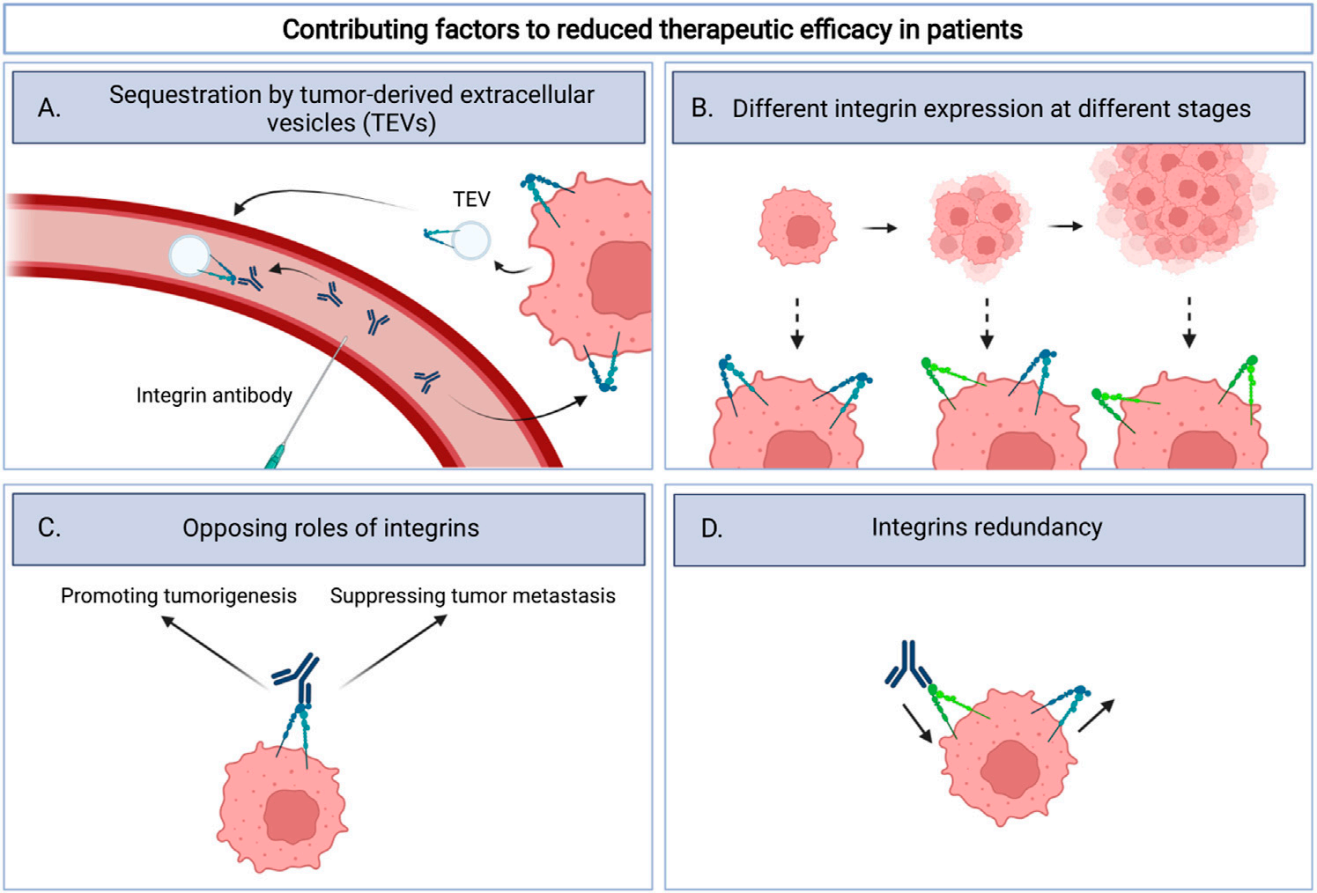
Schematic overview illustrating four factors that could contribute to the lack of clinical efficacy for integrin targeting therapeutics. These include **(A)**. sequestration by tumor-derived extracellular vesicles (TEVs): integrin therapeutics bind integrins on TEVs instead of the tumor itself; **(B)**. Different integrin expression at different stages: integrin expression can change as the tumor progresses and thereby influence target availability; **(C)**. Opposing roles of integrins: Integrins exert tumor promoting effects but may also drive, as yet poorly understood, metastasis suppressing signals. Inhibition of integrins could therefore potentially be disadvantageous; **(D)**. Integrins redundancy: inhibition of one integrin can be compensated by expression of other integrins.

Despite promising preclinical *in vitro* and *in vivo* results that indicate that integrins can be targeted with good efficacy alone, or in combination with radio-, chemo-, or immune therapies, clinical results thus far do not seem encouraging (Goodman and Picard, 2012; Raab-Westphal et al., 2017; Alday-Parejo et al., 2019; Li M. et al., 2021). As with all experimental therapies, recruitment of sufficient numbers of patients fitting the trial design is a challenge. As described above, for one trial this has led to early termination. In addition, testing is often done in the context of advanced disease stages and in cases where earlier therapies have failed. Patients enrolled in the clinical trials described in Table 1 typically have extensive treatment history with the exception of cilengitide that was explored in newly diagnosed glioblastoma patients. This may well explain the discrepancy between clinical trials and results obtained in more acute preclinical models. There are several other factors that may have negatively impacted the clinical testing of anti-integrin therapeutics in cancer. These include variable integrin expression in tumors, redundancy in integrin function, the fact that integrins can have very different roles at distinct disease stages and sequestering of therapeutics by integrin-containing tumor-derived extracellular vesicles (TEVs) (Figure 1).

### Variable Integrin Expression and Poor Pharmacology

Thus far, antibodies have been the major type of anti-integrin therapeutics tested in clinical trials (Table 1). The exquisitely high specificity and corresponding low toxicity of these antibodies are most likely responsible for this high prevalence. A major limitation is a lack of knowledge with respect to expression of the target integrin in the tumor of the patient. Prior treatments may have affected integrin expression patterns in the tumor tissue. In addition, data on antibody pharmacology is generally lacking in the clinical studies. It is well known that targeting of therapeutics to the tumor tissue can be difficult due to poor vascularization, (Carmeliet and Jain, 2011), and this may be a significant problem for the relatively large antibody drugs. Hence, it is important to determine expression of the target integrin and establish actual reach of the integrin-targeting antibodies to the tumor tissue to relate these aspects to response rates in individual patients.

### Redundancy and Different Roles of Integrins at Distinct Disease Stages

Many integrins show overlap in their ligand binding spectrum. I.e., key ECM proteins present in cancer tissues such as fibronectin, laminins and collagens can be recognized by more than one integrin (Danen, 2005). Hence, the effect of blocking one integrin may be compensated for by another integrin binding the same ligand. Patients entering experimental trials often present with a mix of primary and metastatic lesions at different stages. Integrin expression has been observed to differ between primary and metastatic lesions indicating that therapies may affect one but not the other stage. e.g., expression of integrin α2β1 was shown to promote tumor growth of a breast cancer cell line whereas α2β1 expression was attenuated once the breast cancer cells colonized the bone (Moritz et al., 2021). In fact, integrins have been shown in some cases to have opposing roles at different stages and repress rather than support disease progression and metastasis. While depletion of β1 integrins led to reduced outgrowth of primary tumors, it enhanced metastatic capacity in an orthotopic model using triple negative breast cancer cells (Truong et al., 2014). Deletion of β1 integrins also increased prostate cancer progression in a genetic mouse model (Moran-Jones et al., 2012). Likewise, specific deletion of one of the β1 integrins, α2β1, was demonstrated to inhibit tumor metastasis in mouse models for breast or prostate cancer (Ramirez et al., 2011; Moritz et al., 2021). Although similar examples are not described for the αv integrins targeted in clinical trials thus far, these findings suggest that therapeutic targeting of integrins may lead to complex responses in patients that may vary for individual patients.

### Sequestration of Therapeutics by Integrin-Containing Extracellular Vesicles

Another mechanism that may underlie failure of anti-integrin drugs involves TEVs that have been implicated in tumor angiogenesis, immune evasion, and metastasis (Becker et al., 2016). Tumors produce more EVs with a different cargo composition (proteins, lipids and nucleic acids) as compared to normal tissues and these EVs can be derived from the tumor cells as well as other cell types in the tumor microenvironment. Integrins are expressed on TEVs, thus guiding their preference for organ colonization (Hoshino et al., 2015). As integrin expressing TEVs are released by various cancer types they may represent a common obstacle by sequestering integrin-targeting antibodies or peptides before these can reach their tumor target (Fedele et al., 2015; Hoshino et al., 2015; Singh et al., 2016; Carney et al., 2017; Krishn et al., 2019; Li et al., 2020). This concept has also been demonstrated for patients with inflammatory bowel disease where EVs expressing integrin α4β7 prevented vedolizumab from reaching α4β7 expressed on T cells, which may affect therapeutic efficacy (Domenis et al., 2020).

## Integrins as Biomarkers of Cancer Progression

A major challenge for some of the most aggressive tumor types is providing an accurate diagnosis and prognosis for patients suffering from cancer. Integrins may serve as biomarkers in cancer, due to their aberrant expression on tumor cells and cells in the tumor microenvironment (Juliano and Varner, 1993; Danen, 2005; Desgrosellier and Cheresh, 2010; Hamidi and Ivaska, 2018; Cooper and Giancotti, 2019). Recent studies reinforce the idea that some integrins may serve as predictive cancer biomarkers.

### Integrins αvβ3, αvβ5, and αvβ6

Integrin αvβ3 expression has been extensively associated with melanoma progression from an early radial growth phase to an invasive vertical growth and metastasis (Danen, 2005; Desgrosellier and Cheresh, 2010). Recently, differential expression of the integrins αvβ3 and αvβ6 has been observed in two subtypes of prostate cancer. Using patient derived tumor tissue and tumor bearing murine models, αvβ3 was found to be largely absent in prostate adenocarcinoma ADPrCa but significantly upregulated in the more malignant primary neuroendocrine prostatic cancer (NEPrCa) and its metastatic lesions in the lung (Quaglia et al., 2021). Combined with previous findings on the role of αvβ3 in the differentiation of ADPrCa to the aggressive NEPrCa, αVβ3 could have potential as a biomarker in the early detection of this malignant transition in prostate cancer (Quaglia et al., 2020; Quaglia et al., 2021). The expression of integrin αvβ5 has been suggested to represent a predictive biomarker for several cancer types amongst which, breast, hepatic, and gastric carcinomas (Bianchi-Smiraglia et al., 2013; Lin et al., 2018). Recently, elevated levels of αvβ5 have been detected in patients suffering from either glioblastoma or colorectal carcinoma (Zhang et al., 2019; Shi et al., 2021). For both types of cancer, the overexpression of αvβ5 was correlated with an unfavorable overall survival (Zhang et al., 2019; Shi et al., 2021). Integrin αvβ6 has been shown to represent an unfavorable prognostic marker in pancreatic cancer patients (Li et al., 2016). This integrin was recently found to be a promising serum biomarker for patients with pancreatic cancer. Based on the identification of αvβ6 in serum, chronic pancreatic (cP) patients could be distinguished from patients with pancreatic adenocarcinoma (PAC) and high serum levels of αvβ6 were associated with poor survival (Lenggenhager et al., 2021). Up to now, Carbohydrate antigen CA19-9 has been the only biomarker in use for PAC, yet its sensitivity and specificity failed to meet the expectations for use as conclusive diagnostic tool (Goonetilleke and Siriwardena, 2007). A study with a larger patient cohort will be needed to further assess the potential of αvβ6 alone or in combination with CA19-9 as a prognostic serum biomarker for PAC.

### Integrin α5β1

Metastasis in the bones is often lethal in patients with mammary tumors (Coleman, 2006; Wang et al., 2019). Therefore, finding a predictive biomarker is essential for the early recognition of potentially metastasizing tumors. Integrin α5β1 is known for its participation in tumor promoting processes like angiogenesis, proliferation and metastasis (Hamidi and Ivaska, 2018; Hou et al., 2020). In early-stage breast cancer patients, α5β1 expression in the primary tumor was recently associated with the presence of disseminated tumor cells in bone marrow aspirates and poor metastasis-free survival (Pantano et al., 2021). The same study showed that α5 gene silencing or pharmacological inhibition of α5β1 with volociximab attenuated bone colonization following intravenous injection of tumor cells in mice. Hence, stratification of breast cancer patients based on α5β1 expression may represent a way to exploit the potential of α5β1 antibodies, which have thus far not shown clinical benefit. Integrin α5β1 was also found to be upregulated in several gastrointestinal tumors where enhanced expression of ITGA5 corresponded with a poor prognosis (Zhu et al., 2021). Again, these findings warrant larger scale patient studies to explore the potential of α5β1 as a prognostic biomarker in solid tumors.

## Integrin Mediated Drug Delivery

In the area of drug delivery, integrin αvβ3 has been extensively pursued. It represents an attractive target because of its absence from most normal tissues versus expression in tumor tissue, including tumor cells and cells in the tumor microenvironment such as endothelial cells stimulated to undergo angiogenesis (Hood et al., 2002; Arosio and Casagrande, 2016). Integrin binding peptide motifs such as RGD, which binds αvβ3 as well as other integrins, have been incorporated on the surface of drug carrying vesicles (Ruoslahti, 1996). Cyclic RGD peptides (cRGD) have gained interest in recent years given their high binding affinity for αvβ3 (Li N. et al., 2021).

### Liposomal (Like) Drug Carriers

**FIGURE 2:**
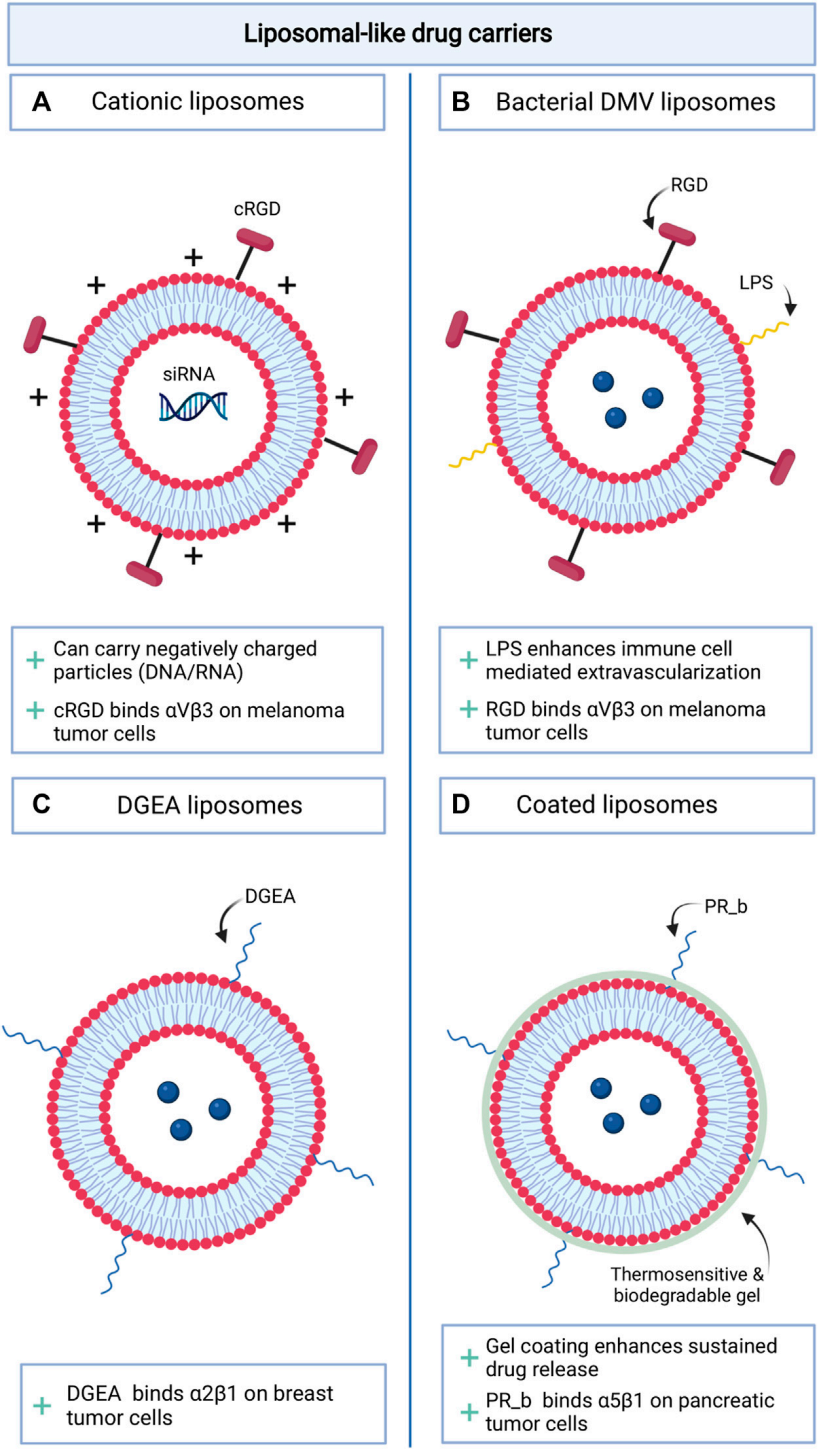
Schematic overview of novel integrin targeting liposomal like drug delivery approaches. **(A)**. cRGD decorated cationic liposomes; **(B)**. Liposomes decorated with a combination of LPS and RGD peptides; **(C)**. DGEA decorated liposomes; **(D)**. Gel coated liposomes decorated with PR-b.

Liposomal vesicles have been used extensively to reduce the toxicity of conventional anti-cancer therapeutics in healthy tissues (Allen and Cullis, 2013). Low treatment efficacy with this approach is caused by ineffective reach of the tumor. The introduction of RGD peptides on the surface of liposomal like vesicles has generally enhanced both drug accumulation in the tumor and anti-tumor efficacy of the drug in mouse models (Fu et al., 2021; Gao et al., 2021; Gong et al., 2021; Khabazian et al., 2021). Additional adjustments were made to the vesicles to further improve their drug transporting characteristics (Figure 2). Sustained drug release of the liposomes was enhanced, making use of PEGylated positively charged lipids (Khabazian et al., 2021). The cationic liposomes decorated with the cRGD peptide were then able to deliver negatively charged siRNA into melanoma cells and effectively induce cell death (Khabazian et al., 2021). Alternatively, Gao et al. developed a double membrane vesicle (DMV), presenting not only the RGD peptide, but also lipopolysaccharides (LPS) (Gao et al., 2021). The association of LPS (normally exposed in the outer membrane of Gram-negative bacteria) with immune cells facilitated the transit of the vesicles from the vasculature into the tumor microenvironment where it could target melanoma cells and deliver therapeutics. Other αvβ3 targeting liposomal like formulations have shown a promising reduction in tumor growth for lung and hepatocellular carcinoma in *in vivo* models (Fu et al., 2021; Gong et al., 2021). Liposomes targeting other integrins are slowly emerging, although selective expression of these integrins in tumor tissue is less evident. Modification of the liposomal membrane with the α5β1 binding peptide PR_b, elevated the tumor specificity of the vesicle for pancreatic cancer cells (Shabana et al., 2021). The addition of a thermosensitive and biodegradable hydrogel in the formulation enabled sustained release of the combination treatment paclitaxel and gemcitabine and attenuated pancreatic tumor growth. Other liposomes presenting the integrin α2β1 binding ligand DGEA, were used to target breast cancer and effectively reduced tumor growth *in vivo* and enhanced the overall survival of the mice (Zhou et al., 2021).

### Alternative Therapy Delivery Approaches

**FIGURE 3:**
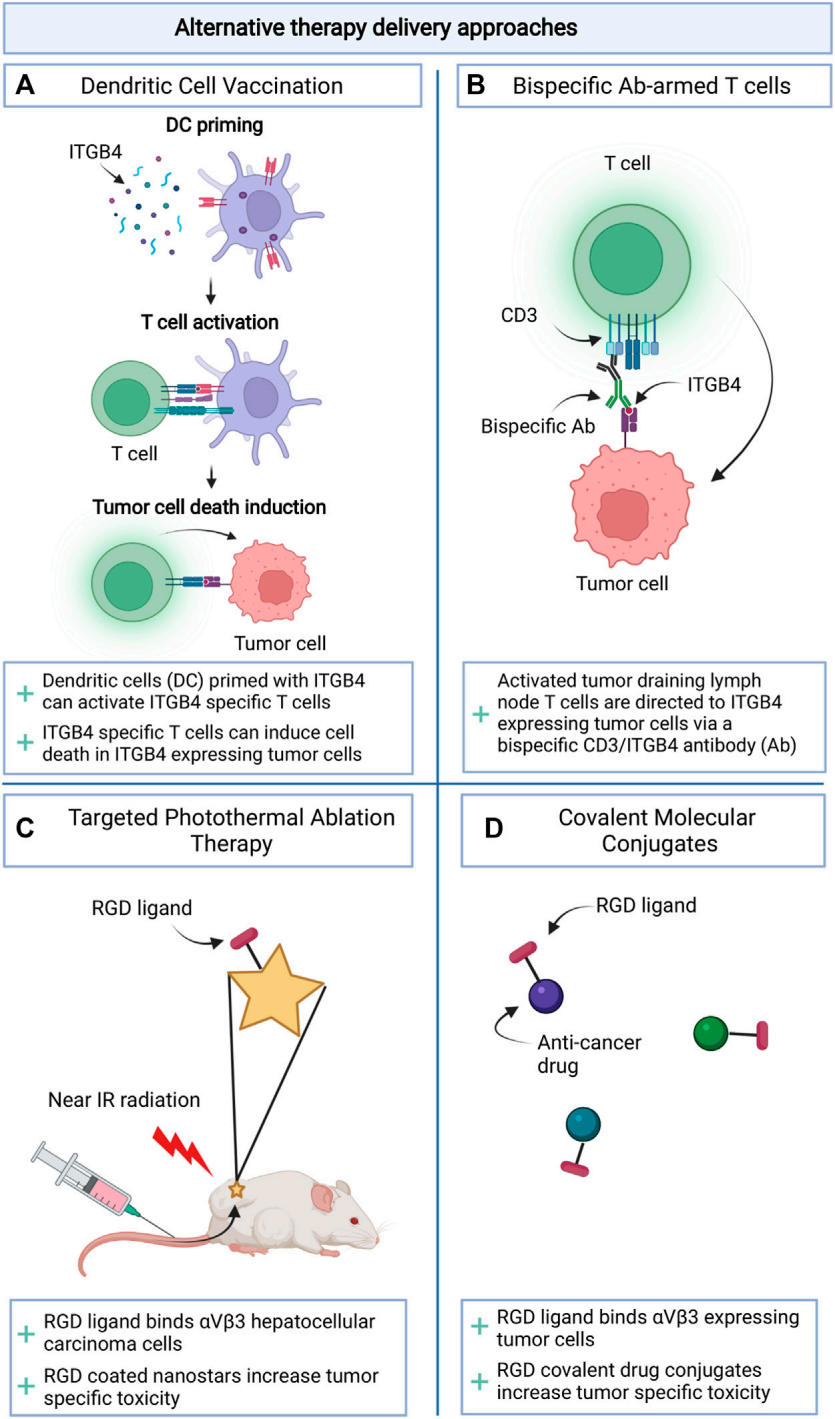
Schematic overview of alternative therapy delivery approaches making use of integrins. **(A)**. Priming dendritic cells for vaccination; **(B)**. Arming T cells with bispecific antibodies; **(C)**. Targeting Photothermal Ablation Therapy; **(D)**. Drug targeting through Covalent Molecular Conjugates.

The use of integrins to direct anti-cancer therapeutics has not been restricted to their application in liposomal drug transport. Integrins may represent targets for the development of novel tumor selective immunotherapies (Figure 3A, B). In mouse models for breast cancer and head and neck squamous cell carcinoma, it was shown that α6β4 is preferentially expressed on CSCs and represents a target for immunotherapies. Vaccination with dendritic cells pulsed with β4 peptide or adoptive transfer of T cells incubated with β4-CD3 bispecific antibodies, could induce T cell anti-tumor activity and inhibition of tumor growth and metastasis formation in the lungs of tumor bearing mouse models (Dobson et al., 2021). The application of covalent linking between an integrin binding peptide (mostly RGD) and an established anti-cancer therapeutic has also been explored (Figure 3D). This approach has led to reduced therapeutic-associated toxicity in healthy tissues (Cirillo and Giacomini, 2021). It will be interesting to compare toxicity profiles for this approach with those of liposomal encapsulations. Lastly, RGD peptides have also been incorporated in polydopamine (PDA) coatings to target photosensitizing agents such as gold nanostars leading to tumor specific cell death and limited adverse effects after near infrared activation of the drug (Li Y. et al., 2021) (Figure 3C).

## Conclusions and Future Perspectives

Thus far, the majority of clinical trials investigating the efficacy of therapeutics targeting integrins in cancer have failed. There are several reasons for these disappointing results, including insufficient insight in the changes in expression of integrins during cancer progression in patients and a lack of knowledge concerning the pharmacological properties and accumulation at the target site of antibodies or peptides. Analysis of these aspects would have to be included in the trial design to understand reasons for failure or success. Other difficulties include the redundancy between different integrins, the different roles that integrins have been found to play at distinct disease stages and sequestration of therapeutic antibodies or peptides by integrins present on TEVs. We envision that 1) further understanding of these hurdles and development of approaches to combat them and 2) incorporation in the trial design of analyses of integrin expression levels and drug accumulation in the tumor tissue should provide avenues for improving therapeutic strategies targeting integrins.

Integrins have been, and continue to be, explored as prognostic biomarkers in cancer, given their stage specific expression patterns. Recent studies further point to their role in distinguishing early-stage low risk-from advanced-stage high risk, metastatic disease. Also, their role as therapeutic targets continues to be investigated. Results thus far do not to point to toxicity as a major issue for drugs targeting αvβ3 and other αv integrins. It will be interesting to monitor the currently ongoing trials exploring α5β1 and αv integrins as targets in various cancers. The recent studies pointing to integrins as targets to attack CSCs, to activate anti-tumor immunity, or to synergize with drugs targeting immune checkpoints suggest exciting new possibilities in this field that await clinical translation. In addition, new strategies exploring integrins as targets for delivery of (liposomes containing) existing anticancer drugs are promising and may contribute to improved targeting of therapeutics and reduced toxicity. Indeed, several exciting possibilities await clinical testing and may well lead to a revisiting of integrins as therapeutic targets.
